# The Effects of Nebulized Inhaled Triptolide on Airway Inflammation in a Mouse Model of Asthma

**DOI:** 10.1155/2023/2983092

**Published:** 2023-08-21

**Authors:** Yafang Miao, Li Wei, Hao Chen, Zeming Zhang, Li Han

**Affiliations:** PCCM, Shanghai University of Medicine & Health Sciences Affiliated Zhoupu Hospital, Shanghai 201318, China

## Abstract

Inhalation of nebulized TP has received little attention in the past. Here, we intend to investigate the effect of nebulized inhaled TP on airway inflammation in a mouse model of asthma. 29 SPF BALB/c mice were divided into four groups: blank control (Blk, *n* = 5), normal saline (NS, *n* = 8), dexamethasone (Dex, *n* = 8), and TP (*n* = 8). During the process of sensitization, mice in the three intervention groups were treated with nebulized NS, an injection of Dex, and nebulized triptolide, respectively. Then bronchoalveolar lavage fluid (BALF), peripheral blood, and lung tissue were collected. Relevant cytokines, transcriptional factors, and CD4+Th17+ T cell proportions were assessed and compared. IL-6, IL-17, IL-23, and TGF-*β*1 demonstrated a significant difference between groups in the following order: Dex < TP < NS (*P* ≤ 0.001), while IL-10 changed in the opposite direction (*P* < 0.001). At the transcriptional level in lung tissue, the Ct value of IL-17 in the Dex group was significantly higher than in the NS and TP groups (*P* < 0.001). Meanwhile, it was higher in the TP group than in the NS group (*P* < 0.001). The Ct value of ROR*γ*t demonstrated a significant difference among three groups in the following order: Dex > TP > NS (*P* < 0.001). An opposite trend of FoxP3 Ct value was revealed in the order: NS > TP > Dex. The proportion of CD4+Th17+ cells was 9.53 ± 2.74% in the NS group, 4.23 ± 2.26% in the Dex group, and 6.76 ± 2.99% in the TP group, which shows significant differences between the NS and Dex (*P* < 0.001) or NS and TP groups (*P* < 0.05). Inhalation of nebulized triptolide can play a role in suppressing airway inflammation with inflammatory cytokines and transcriptional factors reduced and CD4+Th17+ T cells dampened, also in a manner less than injected dexamethasone.

## 1. Introduction

Among all asthma cases, approximately 10% are classified as severe asthma. Current medication regimens, while generally effective, do not control symptoms for a subset of patients [1–4]. Severe cases of asthma are often associated with refractory symptoms, glucocorticoid resistance, and death, at least 50% of which are accompanied by infiltrated or increased neutrophils, rather than the eosinophilic phenotype [4–8]. Previous studies have suggested that airway inflammation dominated by neutrophil infiltration plays a crucial role in such patients [9, 10]. Its mechanism is different from the typical, well-recognized Th1/Th2 imbalance and is more correlated with the imbalance of Treg/Th17 lymphocytes [9, 10], which is generally called a “non-type 2 immune response.”

Triptolide (TP) is a diterpene compound isolated from the thunder god vine *Tripterygium wilfordii*, which has significant anti-inflammatory and immunoregulatory effects. Whether in the form of *T. wilfordii* polyglycoside or single extract, a large number of basic scientific studies have suggested its therapeutic effect in many autoimmune diseases including rheumatoid arthritis and systemic lupus erythematosus [11–13]. Titov and colleagues defined the molecular target of *T. wilfordii*, which lies in the RNA polymerase II (RNAPII) at the core of the transcription factor TFIIH subunits ERCC3 (i.e., XBP) [11]. When TP binds to its target, the specific effect on transcription results in broad inhibition of the immune system. Chinese investigators have also confirmed the anti-inflammatory effects of TP in asthma [12–17]. A cellular culture study showed that TP inhibited airway epithelial goblet cell proliferation and MUC5AC expression via the NF-*κ*B pathway, while airway smooth muscle cell (ASMC) proliferation and migration induced by TGF-*β*1 were moderated through the Smad and NF-*κ*B pathways, suggesting TP's potential therapeutic effect on airway remodeling [12, 14]. In a precedent asthmatic mouse model, levels of white blood cells (WBCs), eosinophils (EOS), IL-17, and IL-23 in BAL were significantly decreased, with mRNA expression of IL-17 in lung tissue also reduced by TP treatment [15]. In another study on human PBMC, the transcription and expression of IL-13 can be reduced by TP by inhibiting the binding of nuclear transcription factors GATA3 and NFAT1 to the promoter, with the effector CD23+ cells suppressed [16]. Moreover, the expression of IL-12/IL-23 could be blocked by TP through activating the CCAAT/enhancer binding protein (C/EBP) in antigen presenting cells (APCs), which enhances the promoter of the common subunit P40 shared by IL-12 and IL-23 [17]. Despite the above activities offered, TP has obvious toxicity and side effects, such as reproductive toxicity, liver and kidney damage, and bone marrow inhibition, which greatly hamper its further clinical application.

Aerosol inhalation is a common mode of drug administration for respiratory diseases. The potential application of nebulized TP in rodent asthma models has received limited attention [12, 14, 15]. In this study, we evaluated the potential therapeutic effects of aerosolized TP in a mouse model of asthma. We investigated its efficacy by observing the anti-inflammatory effect of this innovative dosing route, especially its impact on Treg/Th17 lymphocytes.

## 2. Materials and Methods

In an animal laboratory of Crown Bioscience Taicang (approved by AAALAC), 29 SPF grade BALB/c female mice (6–8 weeks) were divided into four groups (anaesthetized with 3% pentobarbital at 50 mg/kg via intraperitoneal injection, 40 *μ*l/each as average). 5 mice served as no-treatment controls. The asthmatic models established were: 8 mice in the NS group, 8 cases in the Dex group, and 8 cases in the TP group. Then normal saline (NS) atomization, dexamethasone (Dex) intraperitoneal injection, and aerosol TP (using an ultrasonic nebulizer) were administered.

24 mice were intraperitoneally injected with a 400 *μ*l sensitizing solution of 10% ovalbumin (OVA, Sigma, Grade V, ≥98%) with 50 *μ*l of Al(OH)_3_ (aluminium hydroxide adjuvant) 1 mg at 0, 7, and 14 days, respectively. Then 2 mg/ml OVA-NS solution (20 drops each by nasal drip) was given once a day as a stimulus for 5 days continuously from the 21st day. Preceding the stimulation, treatment groups were kept in animal chambers and treated once per day with NS atomization (30 min), Dex intraperitoneal injection 2 mg/kg (250 *μ*l as average), or nebulized TP (NSABE, purity >98%, not derivative, insoluble in water) 2 mg/ml in the solution consisting of double distilled (dd) H_2_O, 5% DMSO (dimethyl sulfoxide), and 30% PEG (polyethylene glycol) with a nozzle fixed upon the face of mice for 30 min.

The mice were sacrificed within 24 hours after the final stimulation by an overdose of anaesthesia (3% pentobarbital at 200 mg/kg via intraperitoneal injection, 160 *μ*l/each as average). The trachea was intubated, and then the left main bronchus was ligated. NS 2.4 ml was used to perfuse the right lung tissue 3 times and then extracted. Bronchoalveolar lavage fluid (BALF) was obtained with a recollection amount of ≥80%. The supernatant was then centrifuged at a speed of 1500 rpm at 4°C for 10 min and frozen at −70°C for measurement of IL-6, IL-10, IL-17, IL-23, and TGF-*β* by ELISA (BioSwamp Life Science Lab, China).

Part of the left lung tissue of mice was resected, fixed with 10% paraformaldehyde, followed by dehydration, paraffin embedding and sectioning, haematoxylin eosin staining, and sealing. The tissue morphology, inflammatory infiltration, and mucosal damage were then observed under a light microscope.

Total RNA was extracted from the tissue of the left lung with Trizol, reverse-transcribed into cDNAs, and used as templates for PCR amplification. The target gene primer sequences are shown in Table 1.

The PCR reaction conditions were: 95°C, 10 min; (95°C, 15 sec; 60°C, 45 sec) × 40; 95°C, 15 sec; 60°C, 1 min; 95°C, 15 sec; 60°C, 15 sec; the experiment was repeated 3 times (ABI 7300, ABI, USA).

Peripheral blood mononuclear cells (PBMC) were isolated from 1 ml of blood from each mouse, adjusted to a density of 1 × 10^6^/L, and added to a 24-well culture plate with RPMI-1640 culture medium. For each well, 1 ml of phorbol ester (PMA, 10 ng/ml), ionomycin (1 *μ*g/ml), and monomectin solution (500 ng/ml) were added and placed in a 5% CO_2_ incubator at 37°C for 4 hours.

First, 0.5 *μ*l of anti-IL-17 Ab was added to the cell suspension, followed by repeated centrifugation, then removing the supernatant, washing and resuspension of the cell pellet, and then incubation at 4°C for 1 hour. After washing with PBS solution twice, flow cytometry (FCM) was used to detect the percentage of Th17+ T cells among CD4+ cells in peripheral blood (Accuri C6, BD, USA).

SPSS13.0 software and R3.6.1 were used for statistical analysis and relevant charts; ANOVA for comparison among groups; and *t*-test for pairwise comparisons (LSD adopted). The measurement data showed as a mean ± standard deviation (SD), statistical inferences made by bilateral tests, and *P* < 0.05 was considered statistically significant.

## 3. Results

### 3.1. Examination of Cytokines from BALF by ELISA

**Figure 1:**
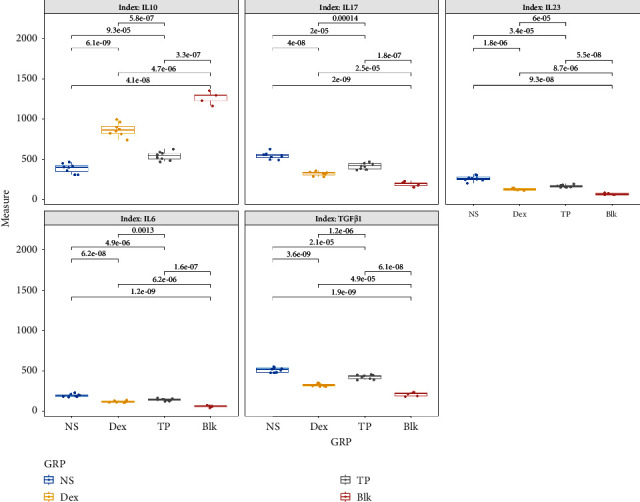
ELISA analysis of inflammatory cytokines in BALF from mice sensitized with OVA and treated with normal saline (NS), the positive control anti-inflammatory Dex, or the test compound TP. Compared with the other two intervention groups, IL-6 was increased significantly in the NS group, and it was decreased more in the Dex group than in the TP group (*P* ≤ 0.001). The anti-inflammatory cytokine IL-10 varied significantly in this order: NS < TP < Dex (*P* < 0.001), while IL-17, IL-23, and TGF-*β* showed variable results, with reduced levels in TP and Dex-treated mice (*P* ≤ 0.001). The units are ng/ml for TGF-*β*1 and pg/ml for the other indexes.

The levels of IL-6, IL-10, IL-17, IL-23, and TGF-*β*1 in BALF were significantly different between the groups (*P* < 0.001). IL-6 of the NS group was significantly higher than that of the Dex and TP groups (*P* < 0.001). And meanwhile, a significant difference between the latter two groups indicated IL-6 was lower in the Dex group (*P* = 0.001). Comparisons of IL-10 showed significant differences between the three groups: Dex > TP > NS group (*P* < 0.001). IL-17, IL-23, and TGF-*β*1 all showed significant differences among the three groups, as follows: Dex < TP < NS (*P* < 0.001) (shown in Table 2 and Figure 1).

### 3.2. Measurement of Key Transcription Factors (TFs) in Lung Tissue

**Figure 2:**
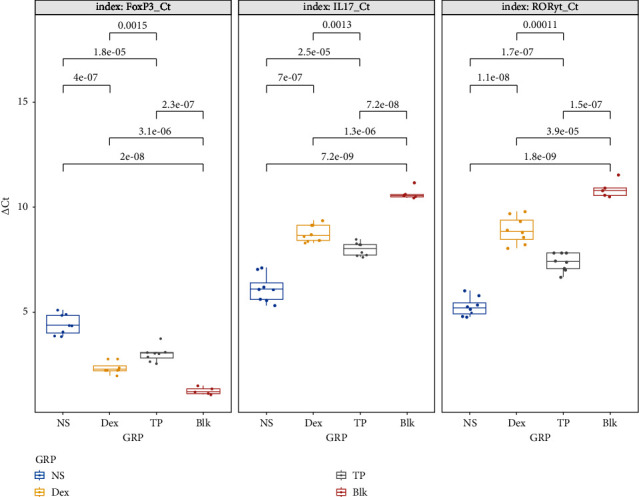
RT-PCR analysis of relevant transcription factors in lung tissue (according to ΔCt between the other TFs and endogenous reference GAPDH). There was significantly higher expression of IL-17 mRNA in the NS than in the other intervention groups (*P* < 0.001). Meanwhile, transcription of IL-17 mRNA dropped more in the Dex group than in the TP group (*P*=0.0013). mRNA expression of FoxP3 in the NS group was significantly decreased compared with the two intervention groups (*P* ≤ 0.001), and FoxP3 transcription in the TP group was lower than in the Dex group (*P*=0.0015). mRNA expression of ROR*γ*t varied in this order: NS > TP > Dex (*P* ≤ 0.001).

∆Ct value of each TF was calculated by subtracting each Ct value from the reference GAPDH. The ∆Ct value of IL-17 in the Dex group was significantly higher than that in the NS and TP groups (*P* < 0.001). There was also a significant difference between the latter two groups (*P*=0.003), suggesting the mRNA level of IL-17 was the lowest in the Dex group (except the Blk group). The ∆Ct value of ROR*γ*t showed significant differences in pairwise comparison, ranking as Dex > TP > NS group (*P* < 0.001), suggesting that mRNA expression of Th17-specific transcription factors was reduced by Dex and TP, with a greater effect of systemic Dex. The ∆Ct value of FoxP3 in the NS group was significantly higher than that of both Dex and TP groups (*P* < 0.001), with a significant difference between the Dex and TP groups (*P*=0.001), which displayed a lower level in the Dex group and the suppressive effect upon the transcription of FoxP3 mRNA (shown in Table 3 and Figure 2).

**Figure 3:**
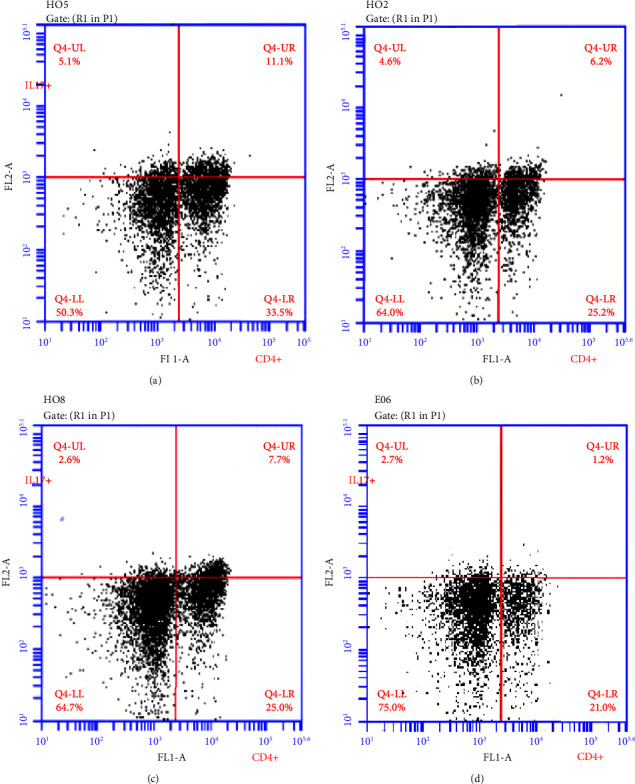
Flow cytometry of Th17+CD4+ in PBMC. (a) NS group, (b) Dex group, (c) TP group, and (d) Blk group. From individual mice, each with 10,000 cells.

**Figure 4:**
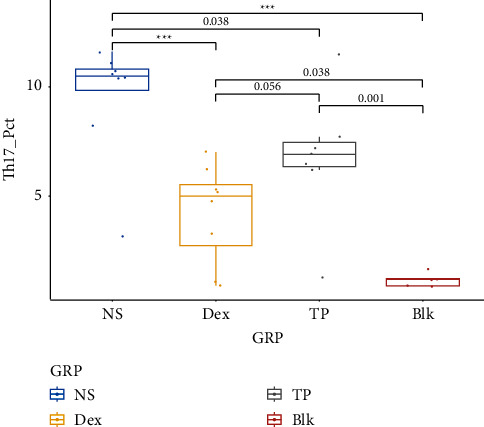
Proportion of CD4+Th17+ expression in PBMC. The proportion of CD4+Th17+ varied in this order: NS > TP > Dex > Blk (*P* < 0.001). The *P* value of difference in pairwise comparison is <0.001 (Dex vs. NS) and 0.038 (TP vs. NS), respectively, indicating Th17 cell numbers were reduced by Dex and TP. The annotations show the statistical results of the LSD *t*-test performed. ^*∗∗∗*^indicates *P* value less than 0.001. Diagram shown as the mean +/− interquartile range.

**Figure 5:**
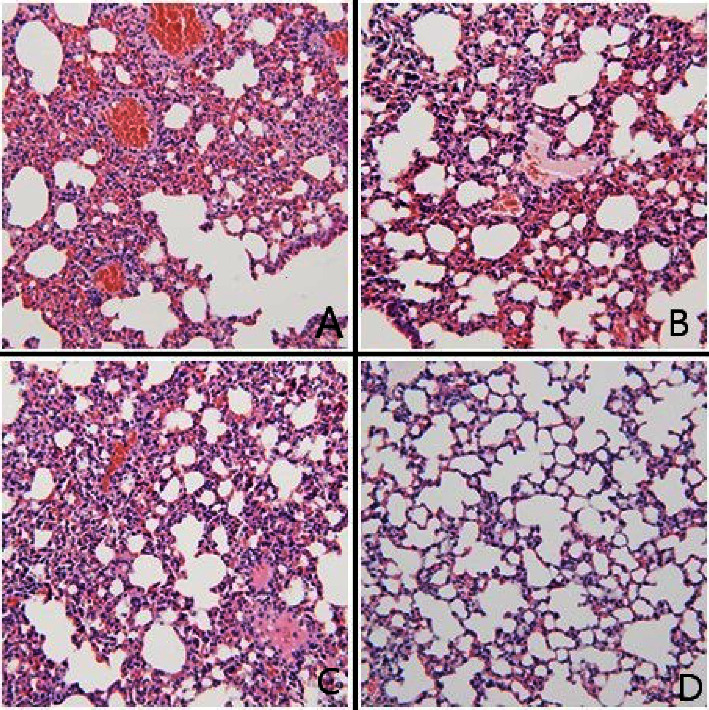
Pathological features of lung tissue in different groups. (A) NS group, (B) Dex group, (C) TP group, and (D) no-treatment control. The images shown are representative of sections from four mice. White blood cell infiltration, thickened alveoli septum, and alveoli filled with secretions (including RBC coagulation in vessels, highlighted with arrow heads) are shown. (HE staining, ×100).

Shown in Figure 3 are the graphs of flow cytometry on CD4+Th17+ T lymphocytes in PBMC from samples of four groups. The proportion of Th17+ was 9.53 ± 2.74% in the NS group, 4.23 ± 2.26% in the Dex group, and 6.76 ± 2.99% in the TP group. A statistical difference existed between the NS and Dex groups (*P* < 0.001), indicating the inhibition of Dex on Th17. It appeared that TP had an apparent down-regulatory effect on Th17 cells compared to NS-treated mice (*P*=0.038). However, there was no significant difference between the TP and Dex group (*P*=0.056) (shown in Table 3 and Figure 4). A significant pathologic difference among the NS group, the treated groups, and the blank group could be found in pairwise comparison (shown in Figure 5), while no significant difference was seen between the Dex group and TP group in terms of pathological scores on lung tissue (by a pathologist).

## 4. Discussion

The results confirmed that nebulized TP has anti-inflammatory activity in our mouse model of asthma. Proinflammatory cytokines such as IL-6, IL-17, IL-23, and TGF-*β* all decreased in BALF due to the effect of Dex or TP. Although the anti-inflammatory effect of inhaled TP was not at the level of i.p. injected Dex, the trend of declining inflammation was consistent. The mRNA of IL-17 and ROR*γ*t (the transcription factor that promotes IL-17 transcription and Th17 cell differentiation) decreased in both the Dex and TP groups (according to Ct values), while FoxP3 (the transcription factor that promotes Treg cell differentiation) increased in both the Dex and TP groups. CD4+Th17+ T cells isolated from PBMC were also reduced in numbers by Dex and TP. The latter could induce the downward trend and reach statistical significance as Dex, though to likely a less extent. Pathological changes in lung tissue also verified the mouse model of asthma and anti-inflammatory effects of TP. Hence, our results provide evidence that TP could attenuate Th17 expression and increase Treg expression simultaneously in this mouse model of asthma.

We are particularly interested in Treg/Th17 cells which are important in the complex pathogenesis of bronchial asthma. Distinct from the traditional hypothesis of Th1/Th2 imbalance, Treg/Th17 cell imbalance is also considered a crucial part of pathogenesis [9, 10]. Th17, a particular subgroup of CD4+ T lymphocytes, can secrete high levels of cytokines such as IL-17A, a process modulated by TGF-*β*, IL-6, IL-21, IL-1, and IL-23, usually under the control of the transcription factor ROR*γ*t. Th17 cells can promote the maturation and differentiation of neutrophils; with their strong chemotactic effect, Th17 facilitates the infiltration of neutrophils in the airway and contributes to the generation of the glucocorticoid-resistant phenotype of asthma. Moreover, Th17 also plays a pivotal role in airway remodeling. Treg lymphocytes secrete anti-inflammatory cytokines (IL-4, IL-10, TGF-*β*, etc.) to mediate immune tolerance (crucial in maintaining the body's immune balance) under the regulation of the transcription factor FoxP3. Both the deficiency of Treg cells and the hyperactivity of Th17 cells are together involved in the immunological pathogenesis of asthma, consistent with previous reports [9, 18].

Although several inflammatory cytokines were quantitated in this study, others such as IL-1*β*, TNF-*α*, and MCP-1 were not included due to a limited volume of samples. Numerous prior studies have shown that a non-type 2 immune response includes the release of several proinflammatory cytokines such as IL-1*β*, IL-6, IL-8, IL-17, and IFN-*γ* [3, 19].

**Figure 6:**
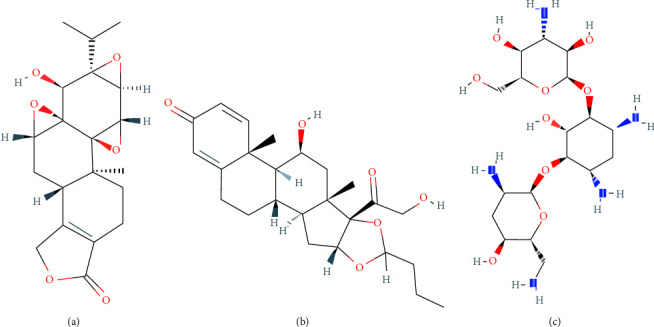
Molecular diagram of (a) triptolide, (b) budesonide, and (c) tobramycin.

Relevant research on asthma has shown that TP has a definite anti-inflammatory effect, but there are yet few reports of using aerosolized TP in animal models of asthma. In a rat model of asthma, nebulized TP can inhibit ASM hyperplasia by down-regulating NF-*κ*B, bcl-2, and PI3K [20], but details of how to instil aerosolized TP into the airway were limited. Triptolide has a molecular formula of C_20_H_24_O_6_ and a molecular weight of 360.4. It is insoluble in water, but soluble in chloroform, dichloromethane, and dimethyl sulfoxide (DMSO), indicating stronger lipid solubility. As a reference, budesonide has been commonly used in clinical practice as an inhaled glucocorticoid for nearly 30 years; its molecular formula is C_25_H_34_O_6_, its molecular weight is 450.3, and it is soluble in both water and dichloromethane. Another drug approved by the FDA for clinical atomization use is the antibiotic tobramycin (molecular formula: C_18_H_37_N_5_O_9_, molecular weight: 467.5), which is soluble in water but poorly soluble in 100% ethanol (molecular diagrams of the above are shown in Figure 6). The three molecules are all alicyclic hydrocarbon structures with 3–5 rings. The smaller molecular weight and stronger lipid solubility of TP may facilitate its entry into the airway epithelium with its anti-inflammatory effects.

The specific binding target of TP is ERCC3 (also known as XBP), the core subunit of TFIIH. Meanwhile, the most related signaling pathway involved in the mechanisms of TP were the NF-*κ*B and MAPK signaling pathways [13]. According to a relatively early research, TP could suppress the dendritic cell-mediated chemoattraction of neutrophils and T cells by inhibiting Stat3 phosphorylation and NF-*κ*B activation [13]. Aldactone can induce the degradation of XBP, which consequently reduces the level of inflammation in the cardiovascular system through regulating NF-*κ*B and AP-1 signaling pathways [21]. Hence, XBP might be a promising therapeutic target for some chronic inflammatory diseases.

## 5. Conclusion

This study revealed that the inhalation of aerosolized triptolide is effective at reducing inflammation in a mouse model of asthma, though less than intraperitoneal injections of Dex. This is a proof-of-concept study which is an initial step for assessing a potential role for TP in asthma treatment. There are still numerous steps to be clarified and optimized, such as the exact molecular mechanism, ideal solvent, suitable dosage, and minimization of toxicity and side effects, all of which merit further investigation. Nebulized medicines have the advantages of direct and rapid action, convenient usage, relatively low doses, and few adverse reactions, so it will be interesting to extend atomized TP inhalation to preclinical studies.

## Figures and Tables

**Table 1:** Target gene primer sequences.

Genes	Primer sequences	Size (bp)
FOXP3	Forward: 5′ GCTCCTCTTCTTGCGAAAC 3″	234
	Reverse: 5′ ACCTATGCCACCCTTATCC 3′	

ROR*γ*t	Forward: 5′ CCATCACTTGCTGCTGTTGTC 3′	120
	Reverse: 5′ TTCACCCAGCCTTTCCCTTTC 3′	

IL-17A	Forward: 5′ GCCTCTGAATCCACATTCC 3′	242
	Reverse: 5′ CTCATCCAGCAAGAGATCC 3′	

GAPDH	Forward: 5′ CTGCCCAGAACATCATCC 3′	197
	Reverse: 5′ CTCAGATGCCTGCTTCAC 3′	

**Table 2:** Changes in relevant cytokines in BALF.

	Blk	NS	Dex	TP	*F*	*P*
IL-6 (pg/ml)	61.24 ± 10.10^*∗*^	196.38 ± 16.05^*∗*^	116.85 ± 10.28^*∗*^	142.01 ± 14.00^*∗*^	116.33	<0.001
IL-10 (pg/ml)	1266.82 ± 71.77^*∗*^	389.43 ± 59.16^*∗*^	868.92 ± 81.23^*∗*^	542.36 ± 53.49^*∗*^	209.52	<0.001
IL-17 (pg/ml)	192.40 ± 28.15^*∗*^	545.71 ± 43.48^*∗*^	322.43 ± 28.34^*∗*^	414.68 ± 39.38^*∗*^	109.07	<0.001
IL-23 (pg/ml)	69.65 ± 11.05^*∗*^	260.42 ± 33.29^*∗*^	126.70 ± 13.80^*∗*^	167.56 ± 15.03^*∗*^	97.14	<0.001
TGF-*β* (ng/ml)	209.61 ± 23.85^*∗*^	512.04 ± 28.76^*∗*^	323.46 ± 17.28^*∗*^	424.80 ± 27.00^*∗*^	177.99	<0.001

^*∗*^Pairwise comparison *P* ≤ 0.001.

**Table 3:** Changes in mRNA transcription of three TFs and expression of CD17+ T lymphocytes.

	Blk	NS	Dex	TP	*F*	*P*
IL-17 (∆Ct)	10.69 ± 0.29^*∗*^	6.19 ± 0.66^*∗*^	8.80 ± 0.41^*∗*^	8.06 ± 0.32^*∗*^	105.95	<0.001
ROR*γ*t (∆Ct)	10.90 ± 0.41^*∗*^	5.32 ± 0.45^*∗*^	8.96 ± 0.65^*∗*^	7.43 ± 0.44^*∗*^	143.33	<0.001
FoxP3 (∆Ct)	1.32 ± 0.18^*∗*^	4.48 ± 0.48^*∗*^	2.42 ± 0.28^*∗*^	3.07 ± 0.36^*∗*^	88.32	<0.001
CD4CD17 (%)	1.18 ± 0.33** ^**	9.53 ± 2.74 ˜	4.23 ± 2.26** ^ ˜**	6.76 ± 2.99** ^ ˜**	13.70	<0.001

^*∗*^Between any two groups in pairwise comparison: *P* < 0.01;  ^either group in pairwise comparison with NS group: *P* < 0.05;  ˜either group in pairwise comparison with Blk group: *P* < 0.05.

## Data Availability

The data used to support the findings of this study are available from the corresponding author upon reasonable request.

## Abbreviations

TP: Triptolide
NS: Normal saline
Dex: Dexamethasone
BALF: Bronchoalveolar lavage fluid
RNAPII: RNA polymerase II
IL: Interleukin
TGF-*β*: Transforming growth factor-beta
PBMCs: Peripheral blood mononuclear cells
PBS: Phosphate buffer saline
GAPDH: Glyceraldehyde-3-phosphate dehydrogenase
ELISA: Enzyme-linked immunosorbent assay
FCM: Flow cytometry
IFN-*γ*: Interferon-gamma
TNF-*α*: Tumor necrosis factor-alpha
TSLP: Thymic stromal lymphopoietin
MIP-3*α*: Macrophage inflammatory protein-3 alpha
GM-CSF: Granulocyte-macrophage colony-stimulating factor
ASM: Airway smooth muscle
NF-*κ*B: Nuclear factor kappa B.
